# Supplementary material for: Neurotrophin Analog ENT-A044 Activates the p75 Neurotrophin Receptor, Regulating Neuronal Survival in a Cell Context-Dependent Manner
Source: Int J Mol Sci. 2023 Jul 20;24(14):11683. doi: 10.3390/ijms241411683 (PMC10380564; doi:10.3390/ijms241411683)
Supplement: Supplementary file 1 [file ijms-24-11683-s001.zip › ijms-2491014-supplementary.pdf]

Supplementary Materials

1. **ENT-A044 cannot activate cell death in untransfected HEK293Tcells. ENT-A044 promotes cell survival in transfected HEK293Tcells, expressing only TrkB receptor or p75NTR and TrkB.**

(a)

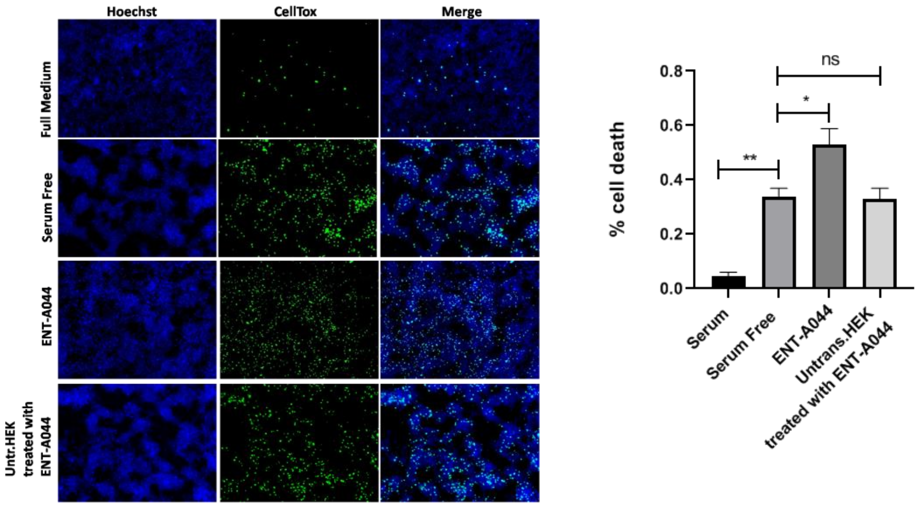

(b)

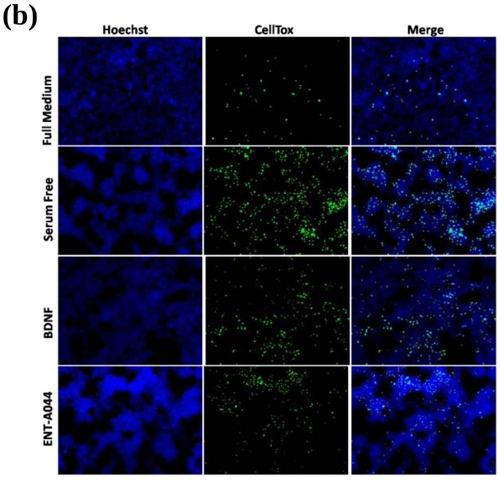

(c)

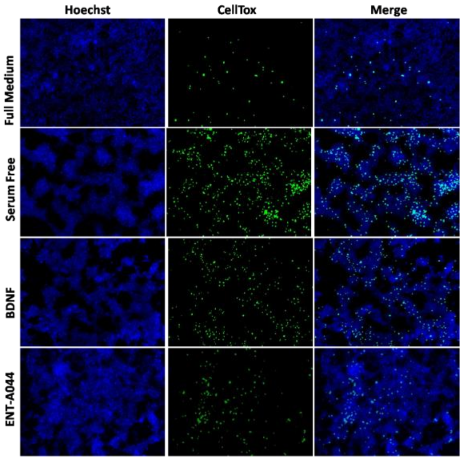

(d)

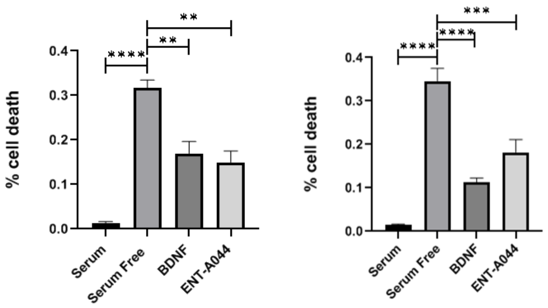

Figure S1. (a) Cell tox assay on transiently transfected and non-transfected HEK293T cells with p75NTR plasmid, after 48 hrs and treatments with the tested compound ENT-A044 (500nM). Quantification of cell tox$^+$ cells (green)/Hoechst$^+$ cells (blue), one-way ANOVA, * $p < 0.05$, ** $p < 0.01$, mean ± SEM of triplicate measurements. **(b)** Cell tox assay on transiently transfected HEK293T cells with TrkB plasmid, after 48 hrs and treatments with the tested

compound ENT-A044 (500nM). **(c)** Cell tox assay on transiently transfected HEK293T cells with TrkB and p75NTR plasmids, after 48 hrs and treatments with the tested compound ENT-A044 (500nM). **(d)** Quantification of cell tox^{+} cells (green)/Hoechst^{+} cells (blue), one-way ANOVA, ** $p < 0.01$,*** $p < 0.005$, **** $p < 0.0001$, mean ± SEM of triplicate measurements.

2. p75NTR and TrkB receptor expression by p7 mouse hippocampal NSCs and human iPSCs – derived NPCs

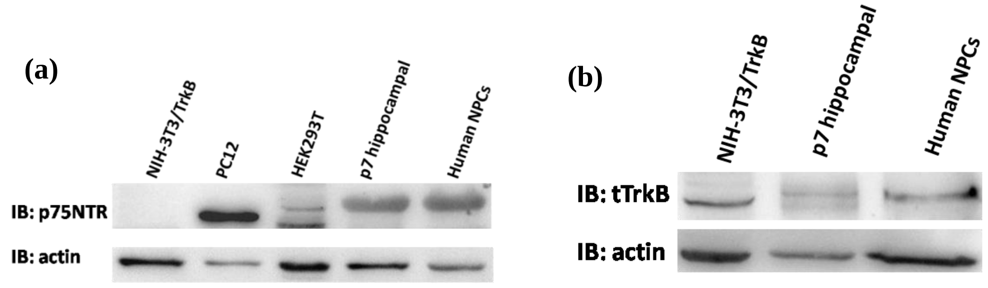

Figure S2. (a) Western blot analysis on lysates from p7 mouse hippocampal NSCs and NPCs that were generated by human induced pluripotent stem cells (hiPSCs), for the detection of p75NTR expression. **(b)** Western blot analysis on lysates from p7 mouse hippocampal NSCs and NPCs that were generated by human induced pluripotent stem cells (hiPSCs), for the detection of total TrkB expression. PC12 cells, stable transfected NIH-3T3 cells and transiently transfected HEK293T were used like controls for p75NTR and TrkB expression.

3. ENT-A044 has no significant effects on p7 hippocampal NSCs after 24h treatments.

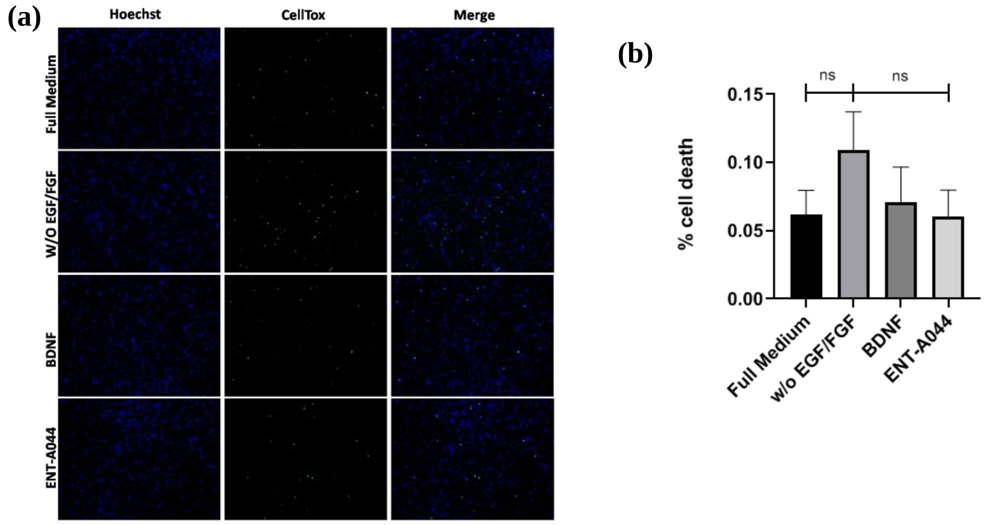

Figure S3. (a) Cell tox assay on p7 mouse hippocampal NSCs after 24 hrs and treatments with the tested compound ENT-A044 (500nM). **(b)** Quantification of cell tox+ cells (green)/Hoechst+ cells (blue), one way ANOVA, ns no significant, mean±SEM of triplicate measurements.
